# Supplementary material for: Charge acceleration without radiation
Source: Proc Natl Acad Sci U S A. 2026 Feb 12;123(7):e2533033123. doi: 10.1073/pnas.2533033123 (PMC12912899; doi:10.1073/pnas.2533033123)
Supplement: Supplementary file 1 — Appendix 01 (PDF) [file pnas.2533033123.sapp.pdf]

Charge acceleration without radiation; Supporting Information

Yakir Aharonov[a,b,c], Daniel Collins[d] and Sandu Popescu[d]
[a] Schmid College of Science and Technology, Chapman University, Orange, California 92866, USA
[b] Institute for Quantum Studies, Chapman University, Orange, California 92866, USA
[c] School of Physics and Astronomy, Tel Aviv University, Tel Aviv 6997801, Israel and
[d] H. H. Wills Physics Laboratory, University of Bristol, Tyndall Avenue, Bristol BS8 1TL
(Dated: Nov 2025)

Appendix A: Acceleration using a capacitor

In most of this paper we use solenoids to accelerate a charged particle without radiation. However we may also use, for example, capacitors, as illustrated in Fig. 5 of main text. Here we explain how that leads to the same effect as with the solenoids.

We start with an electron which is in a superposition of two wavepackets, and place a capacitor between the two. The capacitor is initially discharged, we then charge and discharge it again within a short time T during which the wavepackets, though spreading, do not touch or enter the capacitor. Let $V(t)$, with $V(0) = V(T) = 0$, be the electric potential difference between the capacitor plates. The electric and magnetic fields outside the capacitor are zero at all times, hence, if the electron is prepared in a single wavepacket (to the left or the right of the capacitor) it behaves as it were a free particle and therefore there is no radiation. Then, by the linearity of quantum mechanics, even in a superposition there will be no radiation. Yet a relative phase

$$\alpha = q \int V(t)dt \tag{A1}$$

will accumulate between the two wavepackets. This is easiest to see in the Coulomb gauge where the potential outside the capacitor is constant in space but time dependant. The potential on the left side of the capacitor is $-V(t)/2$, and the potential on the right side is $+V(t)/2$, hence the wavepacket on the left accumulates a phase $-q\int V(t)dt/2$, the wavepacket on the right accumulates a phase $q\int V(t)dt/2$, leading to the phase difference in Eq. (A1).

Therefore we have applied a relative phase between the two wavepackets, just as we did with the solenoids, without causing radiation. A similar situation is if we place each wavepacket in a Faraday cage, and apply a time dependent electric potential difference between the two cages.

Appendix B: Momentum distribution for superposition of two wavepackets

We shall calculate the momentum distribution for the superposition of two wavepackets with relative phase α (in one dimension):

$$\Psi(x) = \frac{1}{\sqrt{2}}\left(\Theta(x+d+a|d) + e^{i\alpha}\Theta(x-a|d)\right), \tag{B1}$$

where Θ is a normalized top-hat function of length L,

$$\Theta(x) = \begin{cases} \frac{1}{\sqrt{d}} & 0 < x \le d \\ 0 & otherwise. \end{cases} \tag{B2}$$

We Fourier transform this into momentum space, which gives

$$\begin{aligned}\tilde{\Psi}(p) &= \frac{1}{\sqrt{2\pi}}\int \Psi(x)e^{-ipx}dx \\ &= \frac{1}{\sqrt{4\pi}}\int \left(\Theta(x+d+a|d) + e^{i\alpha}\Theta(x-a|d)\right)e^{-ipx}dx \\ &= \frac{1}{\sqrt{4\pi}}\int \left(e^{ip(d+a)}\Theta(x|d) + e^{i\alpha-ipa}\Theta(x|d)\right)e^{-ipx}dx \\ &= \frac{1}{\sqrt{2}}\left(e^{ip(d+a)} + e^{-i(pa-\alpha)}\right)\tilde{\Theta}(p|d),\end{aligned} \tag{B3}$$

where

$$\begin{aligned}\tilde{\Theta}(p|d) &= \frac{1}{\sqrt{2\pi}}\int \Theta(x|d)e^{-ipx}dx \\ &= \frac{1}{\sqrt{2\pi d}}\frac{e^{-ipd}-1}{-ip}.\end{aligned} \tag{B4}$$

By rearranging the formula and taking out overall phases, we can rewrite this in the more customary form

$$\tilde{\Psi}(p) = \sqrt{\frac{d}{\pi}}e^{i(pd+\alpha)/2}\cos\left(\frac{pD-\alpha}{2}\right)\mathrm{sinc}\left(p\frac{d}{2}\right), \tag{B5}$$

where $\mathrm{sinc}(x) = \sin(x)/x$ and $D = d + 2a$. The momentum distribution is then

$$\mathcal{P}'(p) = \frac{d}{\pi}\cos^2\left(\frac{pD-\alpha}{2}\right)\mathrm{sinc}^2\left(p\frac{d}{2}\right). \tag{B6}$$

Appendix C: The final state is close to a boosted top-hat

Here we show that the scalar product of the final state of N wavepackets,

$$\Psi_{p_0}(x) = \frac{1}{\sqrt{N}}\sum_{n=0}^{N-1} e^{in\alpha}\Theta(x-nl|d), \tag{C1}$$

with the boosted top-hat function,

$$\Phi_{p_0}(x) = e^{ip_0x}\Theta(x|L), \tag{C2}$$

where $\alpha = p_0L/N = p_0l$, can be made as close to 1 as desired by taking $l \ll 2\pi/p_0$. Their scalar product is:

$$\begin{aligned}\langle\Phi_{p_0}|\,\Psi_{p_0}\rangle &= \frac{1}{\sqrt{NLd}}\sum_{n=0}^{N-1}\int_{nl}^{nl+d} e^{-ip_0x}e^{in\alpha}dx \\ &= \frac{1}{\sqrt{NLd}}\sum_{n=0}^{N-1} e^{in\alpha}\frac{e^{-ip_0(nl+d)} - e^{-ip_0nl}}{-ip_0} \\ &= \frac{1}{\sqrt{NLd}}\frac{e^{-ip_0d}-1}{-ip_0}\sum_{n=0}^{N-1} e^{in\alpha}e^{-ip_0nl} \\ &= \sqrt{\frac{N}{Ld}}\frac{e^{-ip_0d}-1}{-ip_0}.\end{aligned} \tag{C3}$$

Since we approximate p_0x by a step function with each step of length d, we want the phase accumulated in that step, p_0d, to be small. We take $l \ll 2\pi/p_0$, which implies $d \ll 2\pi/p_0$ (since $d < l$). Then the scalar product is approximately

$$\frac{1}{\sqrt{ld}}\frac{(1-ip_0d)-1}{-ip_0} = \sqrt{\frac{d}{l}} = \sqrt{1-\xi}. \tag{C4}$$

Since we have already taken the gap fraction, ξ, to be small, this scalar product can be made as close to 1 as desired.

Appendix D: Moments of momentum do not change under the AB effect

We shall show that if one starts with a superposition of two wavepackets in one dimension,

$$\Psi = \frac{1}{\sqrt{2}}(\Psi_L + \Psi_R), \tag{D1}$$

where Ψ_L does not overlap in space with Ψ_R, and then applies a relative phase α to get

$$\Psi_\alpha = \frac{1}{\sqrt{2}}(\Psi_L + e^{i\alpha}\Psi_R), \tag{D2}$$

then the moments $\langle\Psi_\alpha|\,\hat{p}^n\,|\Psi_\alpha\rangle$ are unchanged for all integer n.

We demonstrate this by computing the moments, and showing that they do not depend upon α. Explicitly:

$$\begin{aligned}&\langle\Psi_\alpha|\,\hat{p}^n\,|\Psi_\alpha\rangle \\ &= \int \Psi_\alpha^*(x)\left(-i\frac{\partial}{\partial x}\right)^n \Psi_\alpha(x)dx \\ &= \frac{1}{2}\int(\Psi_L^* + e^{-i\alpha}\Psi_R^*)\left(-i\frac{\partial}{\partial x}\right)^n(\Psi_L + e^{i\alpha}\Psi_R)dx \\ &= \frac{1}{2}(\langle\Psi_L|\,\hat{p}^n\,|\Psi_L\rangle + \langle\Psi_R|\,\hat{p}^n\,|\Psi_R\rangle),\end{aligned} \tag{D3}$$

where we used the fact that the cross terms between Ψ_L and Ψ_R, such as $\langle\Psi_L|\,(-i\partial/\partial x)^n\,|\Psi_R\rangle$, evaluate to 0 because they have no spatial overlap, since $-i\partial/\partial x$ does not slide $\psi(x)$ to a different spatial location, e.g. to $\psi(x+a)$.

Hence the moments of the momentum distribution do not depend upon α, and so do not change when we apply a non-local AB phase. This argument applies in a similar way if we have many spatially separated wavepackets.
